# Supplementary material for: Nutrient limitation shapes functional traits of mycorrhizal fungi and phosphorus-cycling bacteria across an elevation gradient
Source: mSystems. 2025 Dec 3;11(1):e00523-25. doi: 10.1128/msystems.00523-25 (PMC12817948; doi:10.1128/msystems.00523-25)
Supplement: Supplemental material — Figures S1-S3, plus additional methods related to sequencing and bioinformatics. [file msystems.00523-25-s0001.pdf]

Supplementary Figures

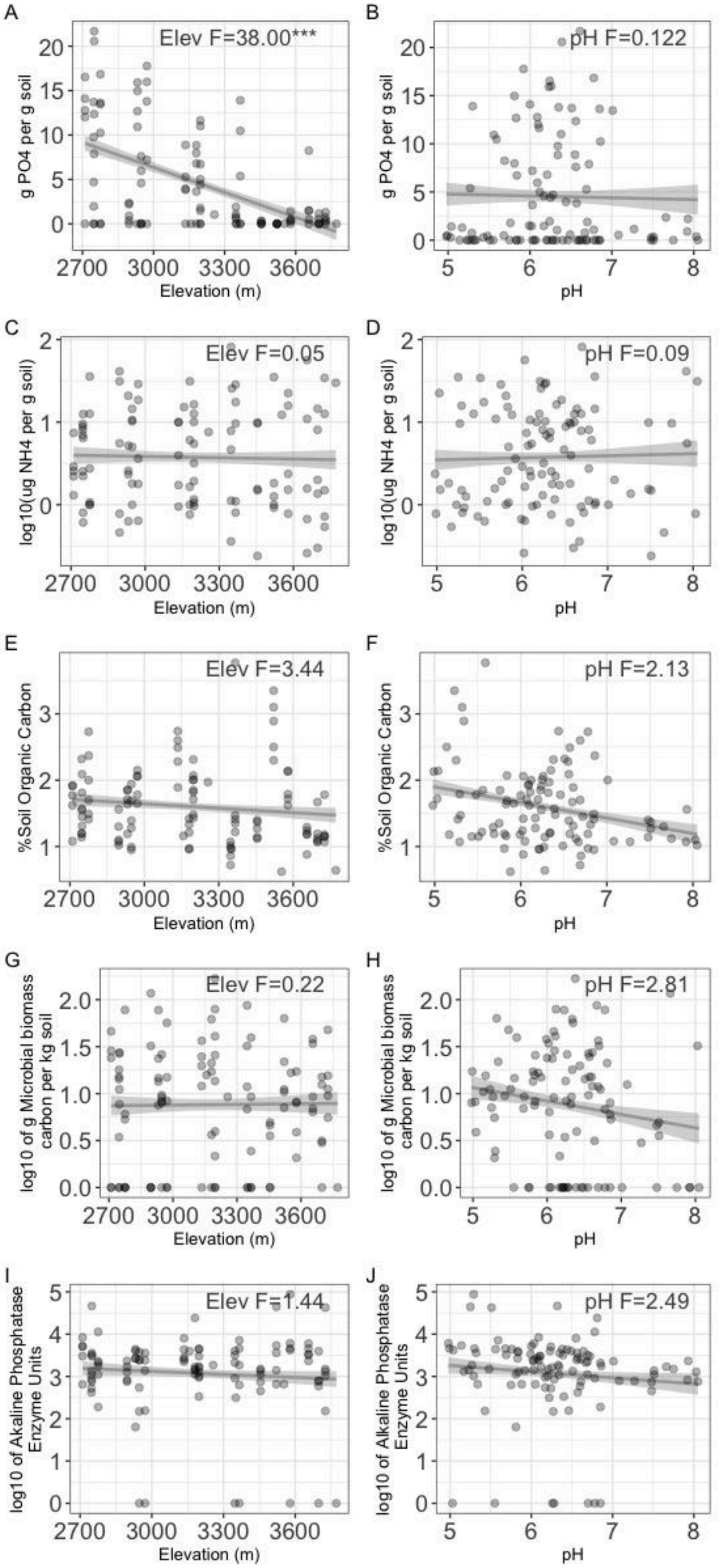

<u>Supplementary Figure 1:</u> Soil Edaphic responses to Elevation and pH. Linear responses of five different edaphic measurements to both Elevation and soil pH. The F-means of either Elevation or pH is shown in the top right corner. F-means were calculated with a mixed-effects linear model accounting for sampling week temporal autocorrelation on (1, 80) degrees of freedom. Stars indicate significance level (* = <.05, ** = <.01, *** =<.001).

<u>Supplementary Figure 2: Recovery of diverse phosphate cycling bacterial genes from alpine meadow soil</u>. Estimated gene copy number for the recovered 22,427 contigs encoding 198 unique P-cycling gene products, which are putatively excreted to the extracellular space and/or have canonical soil P-cycling functions. Data is also included for the rpoB housekeeping gene to serve as an internal control. Each square in the grid represents the mean estimated gene copies for each metabolic pathway and bacterial phylum. Squares also include a point sized by the taxonomic richness of all the genes in each category.

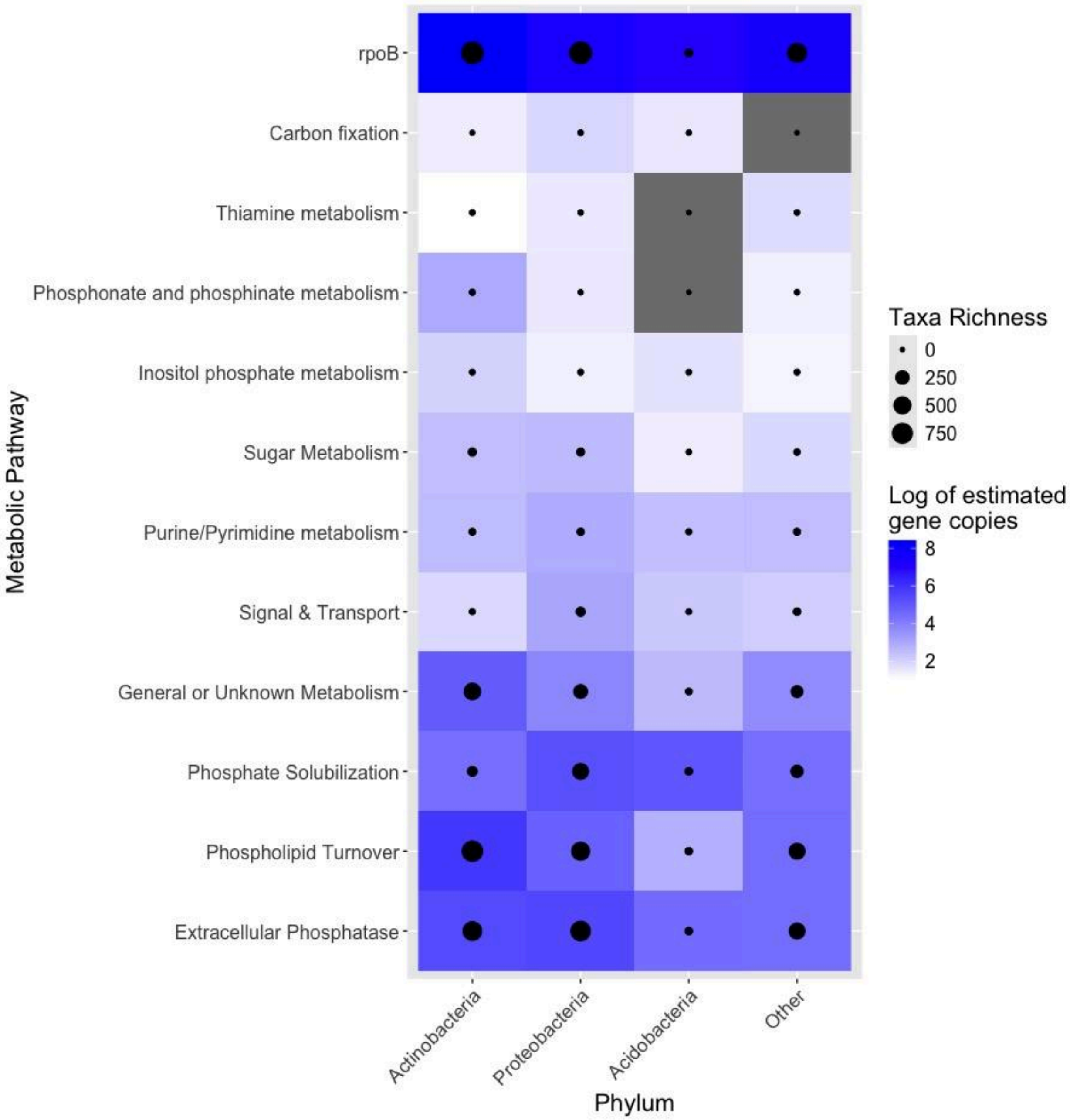

<u>Supplementary Figure 3: </u>: Genus Specific shifts in AMF abundance. Mean cumulative abundance is shown for each AM fungal genus at low, middle, and high elevations.

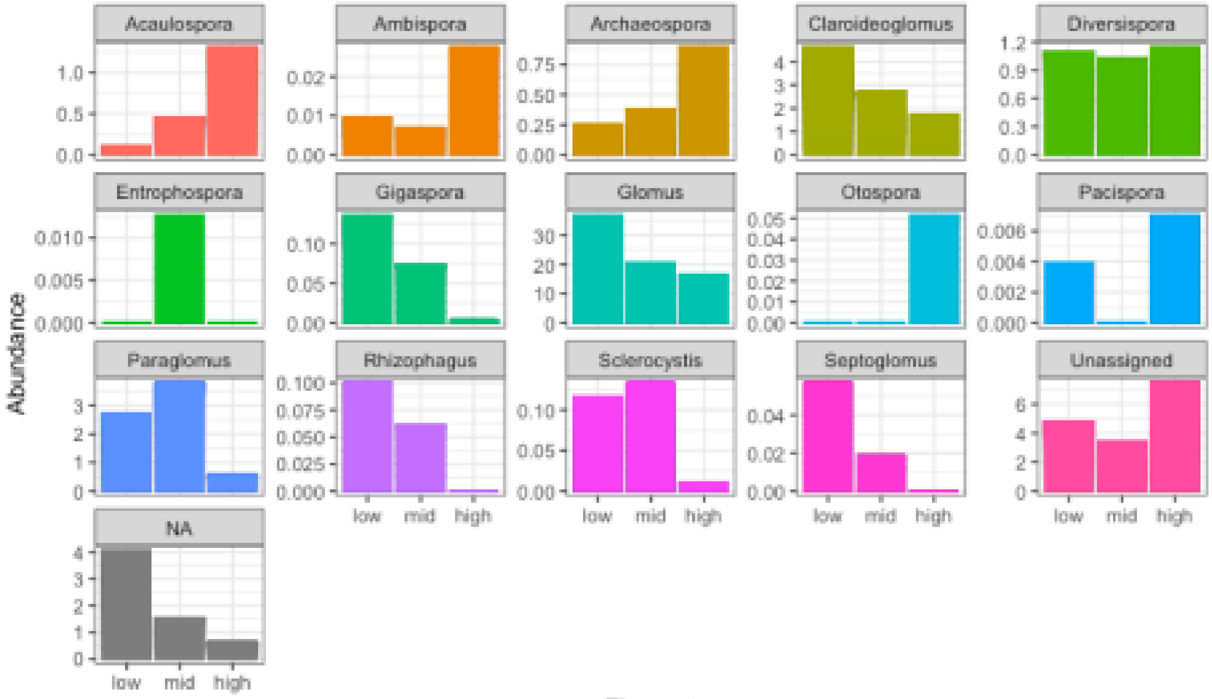

Bioinformatics Appendix

DNA extraction:

We characterized microbial community composition in soil samples that had been processed the same day as collection and stored at -80°C until extractions could be performed. We extracted DNA from soil using a DNEasy PowerSoil kit (Qiagen, Germantown, MD, USA). We followed the manufacturer’s protocol except that we replaced the vortexer step with 2 min in a Mini-Beadbeater (Biospec) and the final resuspension volume was reduced by half to increase DNA concentrations. DNA was stored at -20 °C before use.

AM SSU Marker Gene:

Sequencing: We amplified the most variable region of the small subunit rRNA (SSU) to target arbuscular mycorrhizal fungi using the primer pair NS31/AML2 (Lee et al. 2008). DNA was quantified fluorometrically (Qubit, Invitrogen, Carlsbad, CA, USA) and normalized to 20 ng/l for amplifications. Illumina TruSeq V3 indices (Illumina, San Diego, CA, USA) were used linked to the NS31/AML2 primer pair. Reactions contained 20.5 l of platinum PCR Supermix (Invitrogen, Carlsbad, CA, USA), 1.25 l of each primer (10M), 0.5 l of BSA (20mg/mL), and 2 l of DNA. All PCRs were performed in triplicate with a hot start at 94 C for 3 min, and 25 cycles of 94 C for 45s, 50 C for 1 min, 72 C for 90 s, and a final extension step of 72 C for 10 min. Triplicate PCRs were combined and cleaned with Agencourt AMPure XP magnetic beads (Beckman Coulter, Brea, CA). Our dual-indexed libraries were quantified fluorometrically (Qubit), pooled equimolar, and sequenced on the Illumina MiSeq (2 x 250) at the University of Tennessee, Knoxville core genomics facility.

Bioinformatics: We removed primer sequences using cutadapt. Raw reads were filtered, merged, and ASVs were picked following the DADA2 pipeline [1]. ASVs were taxonomically classified against the Maarjam database [2] . We removed 1021 ASVs that had a cumulative

abundance <0.005% of total read depth [3]. We then center-log normalized read depth using the ANCOM algorithm [4]. Our final dataset contained 648 AM fungal ASVs.

Shotgun Metagenome:

Sequencing: DNA was sequenced at the Joint Genome Institute on the Illumina NovaSeq platform (2 X 150 reads). An Illumina library was constructed using the Kapa HyperPrep library preparation kit. Reads were sequenced using the Illumina NovaSeq S4 platform which generated 59,017,676 reads totaling 8,911,669,076 bp.

Filtering: BBDuk (version 38.86) was used to remove contaminants, trim reads that contained adapter sequence and homopolymers of G's of size 5 or more at the ends of the reads and right quality trim reads where quality drops to 0. BBDuk was used to remove reads that contained 4 or more 'N' bases, had an average quality score across the read less than 3 or had a minimum length <= 51 bp or 33% of the full read length. Reads aligned to common microbial contaminants were removed]. The final filtered fastq contained 58,889,548 reads totaling 8,829,936,278 bp.

Assembly: Base calls from raw reads were corrected using bbcms with bbcms version 38.86. The reads were assembled into contigs using metaSPAdes assembler version 3.14.1[5]. Contigs that are smaller than 200 bp are discarded. The reads were mapped to the final assembly and coverage information was generated with BBMap v38.86.

Annotation: Reads were annotated using the IMG Annotation Pipeline v.5.0.19. Structural annotation to predict noncoding RNA genes (tRNAs, rRNAs, and other RNAs) was performed with tRNAscan-SE v.2.0.6. rRNA genes (5S, 16S, and 23S) as well as other non-coding rRNA genes were identified in contigs using INFERNAL 1.1.3 against the Rfam 13.0 database. Protein-coding genes were predicted with GeneMark.hmm-2 v1.05 and Prodigal v2.6.3. CDSs shorter than 75 bp/ 25 aas are discarded. Protein coding genes were annotated for function with HMMER 3.1b2 against the following databases: SMART 01_06_2016, COG

2003, TIGRFAM v15.0, SuperFamily v1.75, Pfam v30, and Cath-Funfam v4.2.0. To estimate abundance, the contig coverage information is used to calculate “estimated gene copies” by multiplying the average contig coverage by the number of genes in COG, Pfram protein family group, etc. The taxonomic identity of each CDS was determined with lastal 1066. Supplementary Table 5 contains information about the results of the filtering, assembly, and annotation processes. Data are publicly available through the JGI’s data portal under the IMG Genome IDs and Gold Analysis project IDs in sTable 5.

Selection of Putative P-cycling Exoenzymes:

P-cycling genes: Based on KEGG, Enzyme Commission (EC), and PFAM functional annotations, we subset all genes with putative P-cycling functionality (sTable 1). We defined putative p-cycling genes as those classified into metabolism of phospho-organic matter (375 KEGG IDs), having protein structures associated with phosphate mineralization or organophosphorus transformation (456 Pfam domains/families), or interacting with any type of phosphorus bond (1604 EC codes). This initial filtering step yielded ~570,411 contigs containing putative p-cycling annotations for 1,405 unique gene products distributed across 15,168 taxonomic lineages.

Signal peptide detection: Because this group of genes contained mostly basal cellular functions unlikely to impact ecosystem level P cycling, we then subset this group down to P-cycling genes that produce extracellular P-cycling enzymes using signalP [6]. SignalP uses a neural network to detect signal peptides in DNA sequences, indicating that transcription of the gene will be followed by secretion across the cell membrane to the extracellular space. SignalP scored 3938 CDSs as having either Sec/SPI or Sec/SPIII (2510 CDSs), TAT/SPI1 (611), Tat/SPII (52), or TAT/SPII(611) signal peptide sequences. We then manually curated the resulting gene products to remove proteins that would not be relevant to phosphorus cycling

and those involved in extracellular processes not related to nutrient cycling eg chaperone and cell division proteins.

Regardless of signal peptide presence, we also included genes annotated with known ecologically significant P-cycling functions, including alkaline phosphatase, acid phosphatase, phytase enzymes, and genes related to phosphate solubilization through gluconic acid secretion [7]. Our final dataset included 22,427 contigs of 198 unique gene products from 3,718 unique taxonomic lineages. Supplementary Table 2 contains a full list of final recovered P-cyc genes and what metabolic pathway they belong to.

rpoB Housekeeping Gene: We also analyzed all contigs annotated with genes for rpoB. Because rpoB is highly conserved in bacteria, we used it as a 1) marker gene to model P-cycling to overall bacteria composition, and 2) a housekeeping gene to normalize read depths between samples. In each sample, all P-cycling estimated gene copies are represented as relative to the cumulative depth of rpoB.

References:

1. Callahan BJ, McMurdie PJ, Rosen MJ, Han AW, Johnson AJA, Holmes SP. DADA2: High-resolution sample inference from Illumina amplicon data. *Nat Methods* 2016; **13**: 581–583.
2. Opik M, Vanatoa A, Vanatoa E, Moora M, Davison J, Kalwij JM, et al. The online database MaarjAM reveals global and ecosystemic distribution patterns in arbuscular mycorrhizal fungi (Glomeromycota). *New Phytol* 2010; **188**: 223–241.
3. Bokulich NA, Subramanian S, Faith JJ, Gevers D, Gordon JI, Knight R, et al. Quality-filtering vastly improves diversity estimates from Illumina amplicon sequencing. *Nat Methods* 2013; **10**: 57–59.
4. Lin H, Peddada SD. Analysis of compositions of microbiomes with bias correction. *Nat*

Commun 2020; **11**: 3514.

5. Nurk S, Meleshko D, Korobeynikov A, Pevzner PA. metaSPAdes: a new versatile metagenomic assembler. *Genome Res* 2017; **27**: 824–834.
6. Teufel F, Almagro Armenteros JJ, Johansen AR, Gíslason MH, Pihl SI, Tsirigos KD, et al. SignalP 6.0 predicts all five types of signal peptides using protein language models. *Nat Biotechnol* 2022; **40**: 1023–1025.
7. Zeng J, Tu Q, Yu X, Qian L, Wang C, Shu L, et al. PCycDB: a comprehensive and accurate database for fast analysis of phosphorus cycling genes. *Microbiome* 2022; **10**: 101.
